# Attentional capture effects are modulated by the number of concurrently maintained search goals

**DOI:** 10.3389/fcogn.2026.1818391

**Published:** 2026-04-22

**Authors:** Katherine Sledge Moore, Ariel M. Kershner

**Affiliations:** 1 Department of Psychology, Arcadia University, Glenside, PA, United States; 2 Department of Psychology, Neumann University, Aston Township, PA, United States

**Keywords:** attentional capture, hybrid search, memory, RSVP, set-specific capture, visual search

## Abstract

**Introduction:**

In hybrid visual-memory search, multiple search goals are concurrently maintained in memory and searched for in a visual display. When there are few goals, maintenance can operate in visual working memory, whereas when there are many concurrent search goals, they may be maintained in activated long-term memory. A central question here is how multiple search goals are concurrently maintained in memory and selectively enhanced during search. Two attentional capture effects illustrate goal enhancement in the presence of distraction: contingent capture, when distractors resemble the current trial's target, and set-specific capture, when distractors resemble one of your search goals, but not the current trial's target.

**Methods:**

In the present study, participants memorized sets of 2, 4, or 16 objects, and searched for them in a continuous rapid serial visual presentation stream. On critical trials, a goal-related distractor appeared 1–2 frames before the target, matching either the *same* category as that trial's target (contingent capture) or a *different* target category than that trial's target (set-specific capture).

**Results:**

Contingent capture was greatest with 16-item sets, whereas set-specific capture was greatest with 2-item sets, and 4-item sets were in between.

**Discussion:**

These findings indicate that search goal enhancement flexibly adapts to memory load, with implications for models of goal maintenance and attentional control. They also provide the first evidence of set-specific capture with complex objects as search goals.

## 1 Introduction

It's Monday night and you are stopping at the grocery store to pick up ingredients for dinner. As you walk down the aisles, you remember that you need pasta, tomato sauce, garlic, onion, and broccoli. Leading visual search models state that a search template held in memory guides your search, and attention enhances the processing of features in the display that are shared with your target item (Corbetta and Shulman, 2002; Wolfe, 1994), allowing you to easily find the pasta and tomato sauce. But when you get to the produce department, how do you search for garlic, onion, and broccoli? Do you prioritize one at a time? Do you explicitly load a template for “garlic” into visual working memory before onion and broccoli? Or, is your search for these ingredients dictated by the items that may distract you in the produce department? For example, your eyes may land on the cauliflower. Does this distractor result in an enhancement of your search goal “broccoli” due to their similarities, at the expense of your other search goals? Here, we characterize this hybrid visual-memory search using two attentional capture effects: *contingent attentional capture*, in which objects similar to the same search goal influence your search for a target object, and *set-specific attentional capture*, in which objects similar to a different search goal influence your search for a target object. In this example, you have a relatively small set size of concurrently maintained search goals—only those necessary for Monday night's dinner. How do these enhancement effects scale up as you concurrently maintain more search goals, perhaps during your grocery trip to plan for the week?

Similar questions have been asked in the memory and attention literature—how many templates in visual working memory can guide visual search at a time? (Olivers et al. 2011) originally proposed the dual-state hypothesis as a mechanism for guiding search. This hypothesis indicated two functional states in visual working memory (VWM): an active state which serves as a template and interacts with visual attention, and an accessory state which does not interact with attention. This is in line with the single-item template hypothesis (SIT), when only a single item in VWM is in a state that will interact with attention (Bahle et al., 2018). The alternative is the multiple-item template hypothesis (MIT), when multiple items can be maintained in a state that will interact with attention. These hypotheses were put in competition, with observers searching for a target that could be of two different colors (Beck et al., 2012). When the color probabilities were equivalent, participants experienced no switch cost and appeared to simultaneously search for both colors, supporting the MIT. Moreover, the templates were flexible and based on task instructions. Participants could search sequentially if instructed, producing switch costs when changing which template guides attention. Thus, it appears that there may be specific conditions under which multiple templates can guide attention (Duan et al., 2025; Fratescu et al., 2019; Kerzel and Witzel, 2019; Zhou et al., 2020).

Multiple search goals are simultaneously maintained in memory and enhanced when there is a relevant object in visual attention in a hybrid visual-memory search (Cunningham and Wolfe, 2014; Wolfe, 2012). In some hybrid visual-memory search studies, the memory set size is increased up to 100 items, producing a logarithmic, rather than linear, slow in participants' search rate (Cunningham and Wolfe, 2014; Drew et al., 2016; Drew and Wolfe, 2014; Wolfe, 2012). This suggests a logarithmic search through memory during serial visual search across the display to determine if the visual object is in the memory set (Drew et al., 2017). In such a large set size, it is necessary for the search goals to be maintained in activated long-term memory (ALTM) rather than VWM (Cowan, 2001, 2019; Gronau et al., 2024; Saltzmann et al., 2024). Here, ALTM refers to an activated subset of long-term memory that is more available to load into the focus of attention as a search template to compare during the hybrid visual-memory search. If the item in the visual search is not found in the memory search, the system returns to visually searching through the display (Drew et al., 2017). If the item is in memory, the target is selected (Biggs et al., 2015; Drew and Wolfe, 2014; Mitroff et al., 2015; Saltzmann et al., 2024; Wolfe, 2012); however, similar-looking lures can lead to distraction (Gronau et al., 2024; but see Wolfe et al., 2015). What happens when an item in the visual display very closely resembles a search goal in your memory set, but is actually a distractor rather than a target?

Back to the grocery trip example, suppose you arrive home to find that you had purchased broccoli last week and need to use it before it goes bad. You stock the fridge and begin searching for the components of tonight's dinner—garlic, onion, and *last week's* broccoli. However, the new broccoli you picked up today distracts your search, enhancing the “broccoli” search goal. How do we search for a specific target as a search goal in the face of similar distracting objects? One way to quantify this search is through contingent attentional capture, the cost of being captured by a similar object to your search goal before finding the true target of your search (Folk et al., 1992; Luck et al., 2021). We can measure contingent capture costs by comparing search in the presence of a similar distracting object (i.e., same category distractor trials in the current experiment) to search in the absence of a similar distracting object—also referred to as “target alone” trials. This may reveal costs on search of not only the distraction of a similar lure, but also of an enhancement of the target's goal in the priority map (Luck et al., 2021). In contingent attentional capture, one's attention is drawn to a distractor item that bears resemblance to a current target search template. Attentional guidance earmarks the distractor item for further processing, as it resembles the “attentional set” or search goal. This leads the distractor item to be processed in the limited bottleneck for object recognition, causing the participant to experience delays or make mistakes (Folk et al., 2002; Serences et al., 2005).

In your search for *last week's* broccoli, you enhanced the goal of finding broccoli, perhaps to the detriment of your other goals, like finding garlic and onion. What will happen when, after spying the new broccoli, your eyes land on the garlic you need? Here, set-specific capture can quantify the cost that occurs when a goal-relevant distractor and target on a particular trial are not related to the same search goal (Moore and Weissman, 2010). We can measure set-specific capture by comparing search on trials in which the distractor relates to the current trial's target search goal (i.e., same category distractor trials in the current experiment), and trials in which the distractor relates to a different search goal (i.e., different category distractor trials in the current experiment). Just as in contingent capture, after spying the new broccoli, the “broccoli” search goal will be enhanced relative to your other search goals. But what about the “garlic” and “onion” search goals? When a distractor resembling a search goal captures attention, participants shift attention to that goal, enhancing it into the focus of attention at the expense of concurrently maintained search goals (Moore and Weissman, 2011, 2014). In this way, spying *today's* broccoli will distract you and slow your search *more* for garlic than for last week's broccoli because you will have enhanced the “find last week's broccoli” goal at the expense of the garlic search goal. Thus, multiple search goals can be maintained at once, but only one search goal can be enhanced at a time in the focus of attention (Moore and Weissman, 2010, 2011, 2014; Moore and Wiemers, 2018), consistent with visual search models comprised of both parallel and serial components (e.g., Wolfe, 2006) and the MIT hypothesis (Beck et al., 2012). This enhancement in the focus of attention model explains set-specific capture and contingent attentional capture results when searching for two or three items simultaneously, but it has not been tested when there are numerous concurrent search goals.

The current study investigates how multiple search goals are concurrently maintained in memory through the observation of two capture effects. In typical searches for one item at a time, the goal that guides search is likely stored in VWM, allowing it easy access to the focus of attention to enhance that search goal. Items sharing features with the goal stored in the focus of attention serve to capture attention during the search task, slowing the search or causing errors (Downing, 2000; Gronau et al., 2024; Oh and Kim, 2004; Olivers et al., 2006). (Moore and Weissman 2011, 2014) previously characterized search goal enhancement as an automatic, all-or-none process, in which the activated search goal (i.e., the search goal related to a processed distractor) is loaded into the focus of attention so that no other search goal can enter the focus of attention. When you spy the new broccoli while concurrently searching for garlic and onion, the “broccoli” search goal will be enhanced from VWM to the focus of attention relative to “garlic” and “onion,” leading to higher search accuracy when you later see last week's broccoli than when you see garlic (i.e., a smaller contingent capture effect and a larger set-specific capture effect).

The focus of attention model explains set-specific capture and contingent attentional capture results when searching for two or three items simultaneously, but it has not been tested when there are numerous concurrent search goals above the capacity of VWM (Cowan, 2001; Jonides et al., 2008; McElree, 2001; Wolfe, 2020). In this case, it is likely that a form of long-term memory is used to maintain the multiple search goals, such as ALTM (Carlisle et al., 2011; Cunningham and Wolfe, 2014; Hollingworth and Beck, 2016; Olivers et al., 2011; Saltzmann et al., 2024; Woodman et al., 2013). When more than two search goals are concurrently maintained in ALTM, it is possible that goal enhancement would be automatic and uniform, similar to when two search goals are maintained. This would be because the focus of attention could only ever accept one search goal, regardless of the store that the goals were maintained in. If true, the magnitude of the contingent and set-specific capture effects should remain the same, regardless of memory set size.

However, it is also possible that search goal enhancement may be flexible to task demands, such as memory set size. Certain factors, such as experience and practice, may affect search goal enhancement in set-specific capture (Moore and Wiemers, 2018). It is plausible, then, that the magnitude or speed of goal enhancement is affected by the number of concurrently maintained goals. Working memory provides easier access to the focus of attention than does ALTM, which may result in quicker goal enhancement into the focus of attention when there are only two concurrently maintained search goals than many more goals, as both can be retained in VWM simultaneously (Duan et al., 2025; Zhou et al., 2020). At larger memory set sizes beyond the capacity of VWM, goal enhancement from ATLM may be a more sluggish process (Duan et al., 2025; Saltzmann et al., 2024). If so, this could result in an increase of the magnitude of the contingent capture effect, as more search goals are simultaneously maintained (i.e., decreasing accuracy on same-category trials at higher set sizes), because the initial “broccoli” distractor must undergo a more time-consuming recruitment and enhancement of the “broccoli” search goal from ALTM into the focus of attention at a larger set size. For the same reason, set-specific capture effects would decrease as more search goals are concurrently maintained (i.e., increasing accuracy on different-category trials at higher set sizes), because the enhancement of the “broccoli” search goal has not yet overtaken the focus of attention, allowing the subsequent target's search goal to enter the focus of attention. Thus, search goal enhancement would be flexible, changing in efficacy depending on how many concurrently maintained search goals the task demands, as has been shown in the MIT (Beck et al., 2012; Fratescu et al., 2019). This result can speak to the mechanisms of search goal enhancement for set-specific capture and contingent attentional capture, an important question to answer in the capture literature (Gaspelin et al., 2015, 2017; Luck et al., 2021; Mounts, 2000; Sawaki and Luck, 2010, 2013).

In the current investigation, we explored these possibilities by asking participants to memorize sets of two, four, and 16 specific objects. Participants then searched for the memorized set of objects in a continuous rapid serial visual presentation (RSVP) stream, in which any of their memorized objects could appear at any time. On *target alone* trials, a target appeared embedded in a display of unrelated filler objects. On *same category distractor* trials, a distractor resembling that target item appeared just ahead of the target; these trials indexed contingent attentional capture. On *different category distractor* trials, a distractor resembling one of the targets in the search goal set appeared ahead of a different target; these trials indexed set-specific capture. When they completed one block, participants memorized a new set of objects of a different search goal set size and searched for these. If the uniform enhancement hypothesis is supported, we expect capture effects to be the same for each set size. However, if the flexible enhancement hypothesis is supported, we expect increased contingent capture effects and decreased set-specific capture effects with greater set sizes.

In addition to refining our knowledge of the basic mechanisms of visual search and distraction, this investigation has practical significance in that it examines multi-target search, which is common in everyday life. Finally, this is the first investigation to observe set-specific capture using images as distractors (as opposed to a single feature like color). As the results show, the method may be useful in other attentional capture studies that would benefit from the use of images as opposed to isolated features such as colors.

## 2 Methods and materials

### 2.1 Participants

Thirty-seven Arcadia University students aged 18 to 30 years participated in exchange for course credit or $10/h, rounding up to the nearest half hour, usually resulting in a $25 or $30 payout. Seven students were eliminated due to sub-par performance (see Section 3). Based on previously published hybrid search data (Drew and Wolfe, 2014), we estimated that we would need seven participants in order to achieve 90% power in observing differences in search speeds across search goal set sizes (2, 4, and 16), and 17 participants to differentiate between a linear vs. logarithmic growth function for set size. Also based on our prior investigations of set-specific capture, we estimated that we would need 10 participants in order to observe set-specific capture at 90% power, and 24 participants in order to observe contingent attentional capture at 90% power. We aimed for 30 participants in this study as a conservative measure to investigate the interactive effects of distraction (set-specific capture and contingent attentional capture) and search goal set size. All participants reported normal or corrected vision. The study was approved by the Institutional Review Board at Arcadia University and all APA ethical guidelines were followed. Participants provided written informed consent before starting the experiment.

### 2.2 Apparatus and stimuli

The experiments took place on a 20″ or 22″ LCD monitor with a 60 Hz refresh rate, controlled by a PC running Windows 7. Psychtoolbox (Brainard, 1997; Kleiner et al., 2007), implemented in MATLAB, displayed the stimuli and collected responses. The Psychtoolbox code for generating stimuli and the stimulus files can be accessed through the Open Science Framework here: https://osf.io/2vbew/. Participants sat at an unrestricted viewing distance of about 57 cm from the monitor, at which 1 cm on the monitor corresponds to 1° of visual angle, and were told to maintain this distance throughout the experiment (supervised by the experimenter). The room was kept dark during testing.

In the main experiment, participants searched for images of specific objects appearing in a continuous RSVP stream. The objects were taken from the “massive memory” project databases (Brady et al., 2008; Konkle et al., 2010). The database of object categories included 17 objects within each of 200 categories (Konkle et al., 2010). We selected targets (22 in total) and their corresponding distractors from the object categories according to the following criteria: (1) nine of the objects in the category must appear similar to each other, yet still be discernable at rapid presentations; (2) the objects must not be symmetrical—one should be able to answer consistently whether the object is “facing” to the left or to the right^1^; (3) the objects must not have any remarkable emotional valence (e.g., no guns).

**Figure 1:**
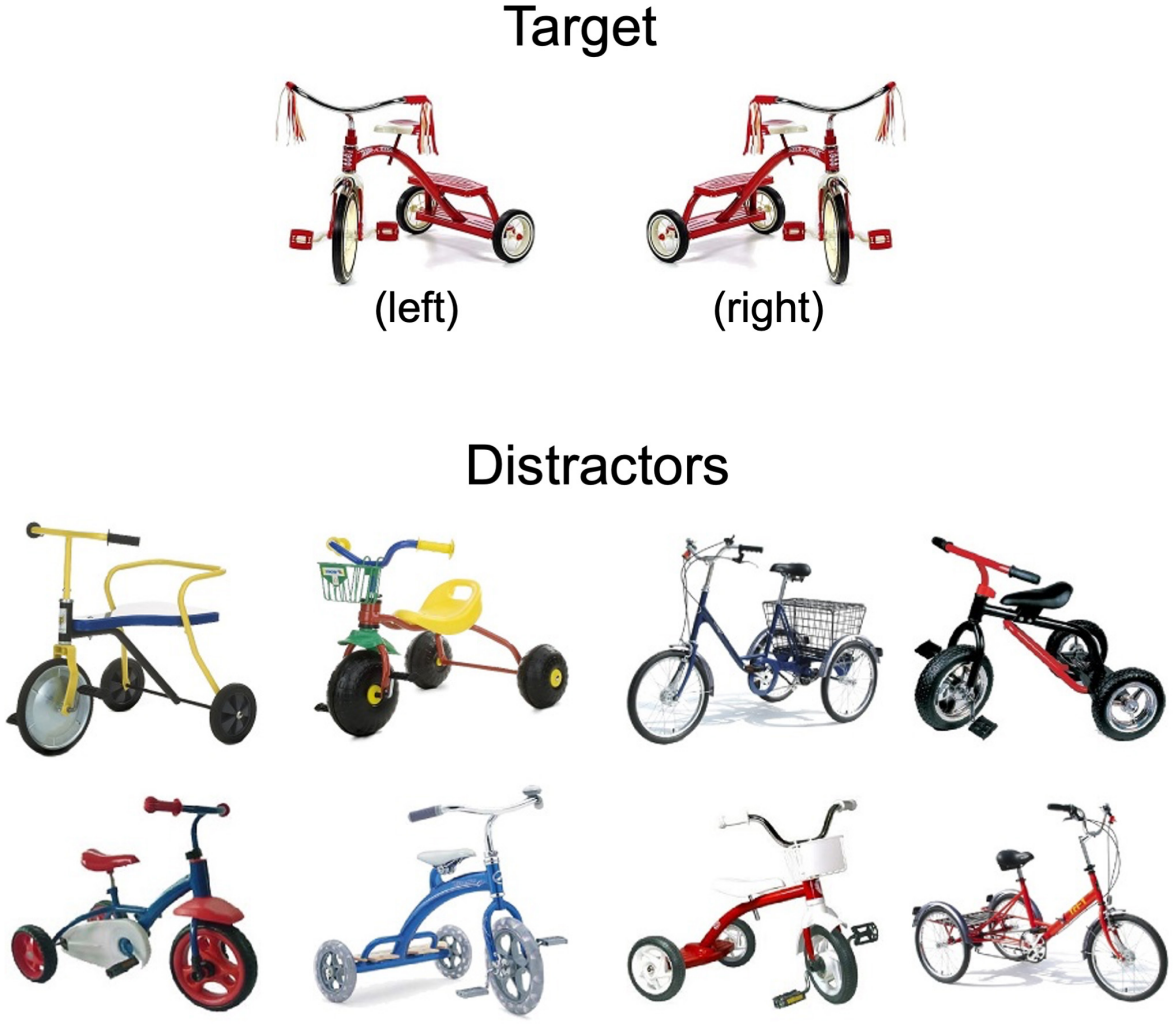
Example target and distractors. Each target stimulus had eight corresponding distractors from the same category that were similar in appearance. Participants judged whether the target was facing to the left or to the right in the search task. All distractors could appear facing to the left or to the right. Distractors always faced in the opposite direction of the target on the same trial.

The object categories we chose were airplanes, cribs, tricycles, bathing suits, birds, cameras, Christmas stockings, coffee mugs, flags, gloves, hairdryers, laptops, turtles, binoculars, staplers, toy horses, trumpets, benches, lawnmowers, flashlights, cell phones, cooking pans, and tennis racquets. In each category, we chose one target item and eight distractors from the 17-item set in the database, choosing the distractors so that they were mildly confusable with the target. For the cribs, bathing suits, coffee mugs, laptops and trumpets, we made slight changes to the coloring of the original stimuli in order to ensure that the distractors were mildly confusable with the target. For all target and distractor objects, we made a left-facing version and a right-facing version by creating a mirror-reversed copy of each image (see Figure 1 for an example of a target and its eight distractors).

In the RSVP display during the search task, most of the images that appeared were “filler” images. We chose our filler objects from the same research group's database that included 2,400 unique objects (Brady et al., 2008). We eliminated any objects that appeared too similar to the targets or that conveyed emotional significance, such as weapons. However, we did not restrict the stimuli to asymmetrical items that face left or right; filler objects included symmetrical items like balls, donuts, etc. We used 2,000 images in total.

There were two tasks to complete in the experiment—the memory “training” task, and the search task. In both tasks, the object stimuli subtended 2.5° × 2.5°.

### 2.3 Procedure and design

Each participant completed a memory training task (to memorize the to-be-searched-for objects) and a search task (the main experiment) for groups of two, four, and 16 objects at a time.

First, participants learned the object identities through the memory training task. During the study period, each target image appeared on the left side of the screen, with the corresponding eight distractor images appearing on the right side of the screen at the same time. Two targets (16 distractors) appeared at a time. Participants were instructed that the targets were on the left and that the objects on the right were *not* targets, and that they should memorize the targets and be able to distinguish them from the distractors presented on the right. Participants were given as much time as they needed to study the target objects before advancing the screen to the next set of two targets to memorize. Once all targets had been viewed, one more display included all target objects together (with no distractors), which participants could study for as long as they wished.

After the self-paced study period, participants were tested for their memory of the target objects. We used a procedure similar to (Drew and Wolfe 2014) and (Wolfe 2012). Participants saw two objects—one above fixation and one below fixation—and were instructed to press an arrow key (up or down) to indicate which object was a target. One of the two objects was always a target and the other was not. Participants completed one block in which each target was paired with an unrelated object (i.e., filler item). Next, the targets were paired with each of the corresponding distractors, providing eight trials per target item. These trials were interleaved and presented randomly (e.g., one trial could include a camera target and its corresponding distractor, the next trial a laptop and its corresponding distractor, and then a camera again with a different distractor). At the end of these two blocks, participants advanced to the next phase of the experiment if their performance was 90% or higher for each target. If not, they repeated the training for just the targets that did not meet the performance threshold until their performance was at or above 90% for all targets. Every participant reached criterion on the first attempt for each set size except for one participant, who needed two attempts to reach criterion in set size 4 only. Overall, the average accuracies on the memory tests across all participants were high and equivalent across set size: 98.7% (set size 2), 99.5% (set size 4), and 99.3% (set size 16).

After completing memory training on a particular set of objects, participants completed the search task. In the search task, participants sought targets from the memorized set in an RSVP stream full of filler objects. When they spotted a target, they indicated whether it was facing to the left (press “J”) or to the right (press “K”). In the instructions and during breaks, participants were reminded about what constitutes facing to the “left” or “right” with example images of each target.

The RSVP display was continuous, which means there was no indication as to when one trial began or ended, or which target may appear on each trial, a method that achieves low floor performance (Moore et al., 2018; Moore and Weissman, 2010, 2011; Serences et al., 2005). Between 18 and 25 filler objects appeared between target items (i.e., from trial to trial). The exact number of filler items varied randomly, so that the timing of each target appearance was unpredictable. This range was selected so that even with a rapid display speed (e.g., 50 ms per item), there was sufficient time for the participant to make a response to one target before the next one appeared. After every 32 “trials,” (target presentations), there was a self-paced break.

The search task started with a practice phase. The time each stimulus appeared on the screen differed depending on the number of items sought, starting at 200 ms for set size 2 and 220 ms for set sizes 4 and 16. The search speed remained at this pace for the first 16 trials so that participants could acclimate to the instructions and task. Participants repeated the practice phase if they performed poorly (below 50% accuracy). Four participants repeated the practice session of their first block (i.e., the practice block of the first set size searched) of the whole experiment before moving on. No participant needed to repeat practice for their second or third set size blocks.

Next, participants completed the calibration phase. In this phase, the search speed changed depending on participants' performance with the goal of determining an RSVP speed at which that participant achieved 75% accuracy, using a staircase procedure. After three consecutive correct responses, the speed was increased [i.e., the inter-stimulus interval (ISI) decreased]. After every incorrect response, the speed decreased (i.e., the ISI increased). The steps up and down were larger (40 ms) at first, to quickly approximate the participants' range. Subsequent adjustments were smaller (down to 5 ms), to more precisely gauge the participants' ideal speed.^2^ There were 64 trials in the calibration phase. The staircase procedure allowed us to match the baseline target alone (TA) performance across participants within each set size. Thus, we can conclude that differences in performance are due to set size and the speed of searching through memory, rather than to difficulty in processing the distractor item (Ghorashi et al., 2003; Nielsen and Andersen, 2011; Robertson et al., 2013).

We calculated the participants' final speed to be the average speed across the last three reversals. Typically in a staircase procedure thresholding experiment, half of the trials start above threshold, with downward adjustments based on performance, and half of the trials start below threshold, with upward adjustments based on performance, and these trial types are interleaved. In this case, because the trial sequence was continuous, it was not possible without choppy speed changes to adjust the speed in two directions, which would also signal trial breaks. Therefore, we opted to start off in the easier, slower direction, and adjust speeds to be faster as participants achieved good performance.

**Figure 2:**
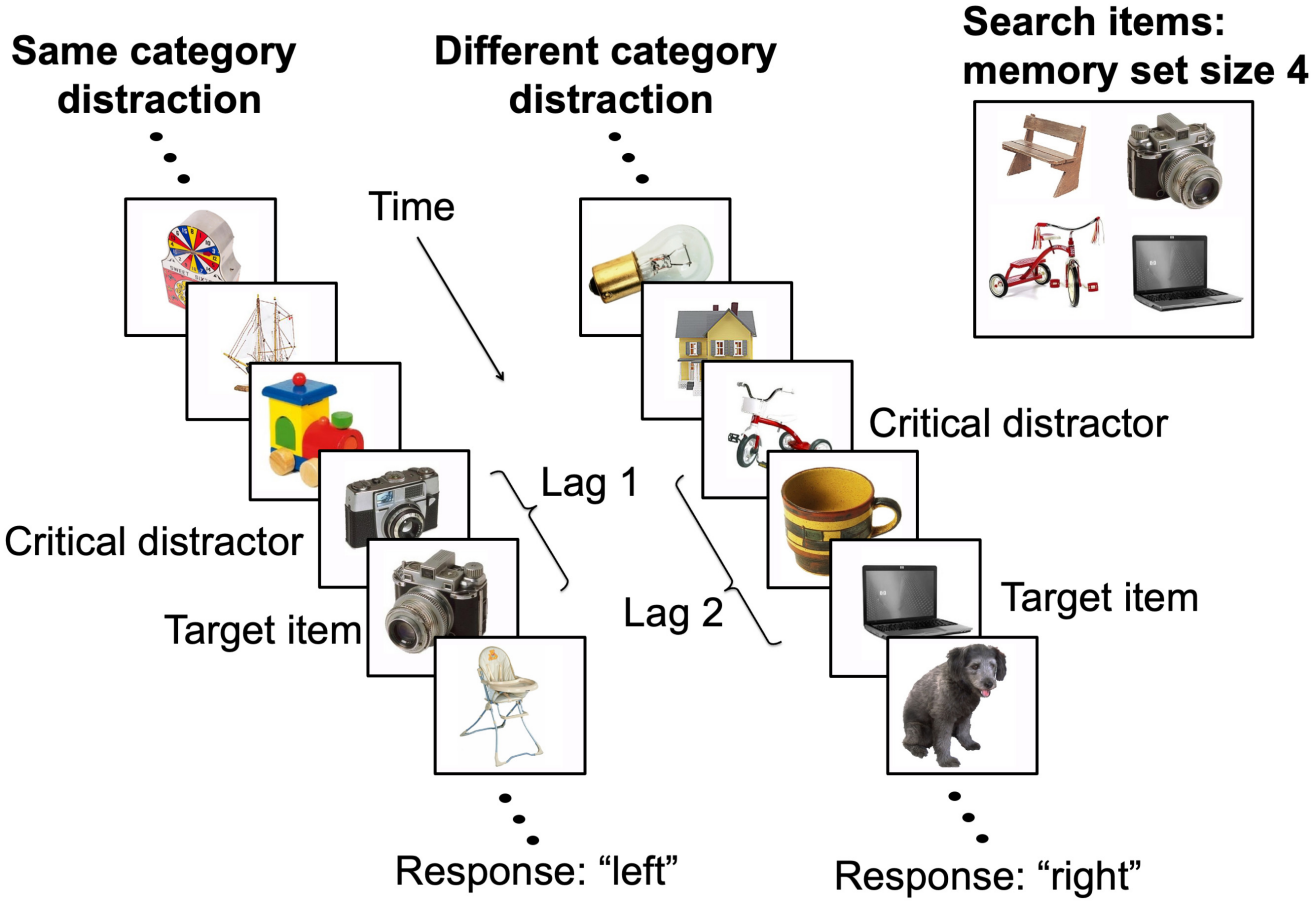
Task schema. The single RSVP display was continuous, and the speed calibrated to the participant and set size. In this example, the participant was looking for a bench, a camera, a tricycle, and a laptop. In the “same category distraction” condition, a stimulus resembling a target (camera) appeared ahead of that target. In the “different category distraction” condition, a stimulus resembling a target (tricycle) appeared ahead of a different target (laptop). Distractors could appear immediately before a target (lag 1) or separated by a filler item (lag 2). In “target alone” trials (not pictured), targets appeared without critical distractors preceding them.

After calibration, participants completed the main search task. In the main search task, there were three trial types. First, there were “target alone” (TA) trials, which participants experienced during the practice and calibration phases. In these trials, targets appeared without any search goal-related distractor objects appearing close in time to targets. Second, there were “same category distraction” (SC) trials. In these trials, a distractor from the same object category as the target appeared ahead of the target. For example, when a participant was looking for a camera, bench, tricycle, and a laptop, a distractor camera appeared prior to the target camera. Third, there were “different category distraction” (DC) trials. In these trials, a distractor appeared prior to the target, but the distractor mapped onto a different search goal. For example, a distractor tricycle might appear prior to a target laptop. For both the SC and DC trial types, distractors appeared either in the frame immediately preceding the target (lag 1), or in the frame two items preceding the target (lag 2; see Figure 2 for examples).

We used lags 1 and 2 to maximize distractor effects. One previous investigation of this kind found that distraction effects were greatest at lag 1 (Moore and Weissman, 2010). However, when distraction appears in a central RSVP, there can be lag-1 sparing effects, in which performance is better when the target immediately follows a distractor (or a second target immediately follows a first target, as in the case of the attentional blink) (Dux et al., 2014; Hommel and Akyürek, 2005). Thus, we included both lags 1 and 2 in order to ensure maximum distraction effects. While doing so, we also tested whether lag-1 sparing effects occur with set-specific capture when a spatial shift is not required, which had not previously been examined.

For all trials with distraction, the critical distractor was facing the opposite direction from the target. Thus, responding to the distractor would yield an incorrect answer. The presentation speed was dependent upon the results from the calibration procedure. For example, the average presentation speed across participants for set size 2 was 96 ms. At that speed, each image was presented for 96 ms and was immediately replaced by the next image with no white space between. However, one participant may have had a presentation speed of 54 ms for set size 2, and another participant may have had a presentation speed of 112 ms.

In the main experiment, the trial distribution was as follows. For set size 2, there were 48 target alone trials, 48 SC trials (12 per target per lag), and 48 DC trials (12 per target per lag). For set size 4, there were 64 target alone trials, 64 SC trials (eight per target per lag), and 64 DC trials (eight per target per lag). For set size 16, there were 96 target alone trials, 96 SC trials (two per target per lag), and 96 DC trials (two per target per lag). There were more trials in set size 16 than set size 2 (and an intermediate number for set size 4) to ensure sufficient number of trials for each target/search goal.

Each participant completed the task (memory training, calibration procedure, and main experiment) for each of the three search goal set sizes, with the order varied so as to fit the testing into two 1–1.5 h sessions. Participants completed set size 16 in one session, and set sizes 2 and 4 in another session. The order of set sizes 2 and 4 was counterbalanced, as was the order of which session (16 or 2 and 4) occurred first. There was no restriction for when the sessions could be scheduled; some participants completed them back-to-back, with a brief (5 min minimum) break between the sessions, whereas others completed the sessions up to 3 days apart.

## 3 Results

We eliminated seven participants whose target alone performance during the main experiment fell below 0.4 proportion correct in any search goal set size, which was far less than the 0.75 proportion correct aimed for in the calibration procedure. Such a performance drop indicated that participants lost attention during the main experiment, or that the calibration procedure did not work for that participant. We accepted other performance fluctuations above and below 0.75 proportion correct on target alone trials. This performance threshold yielded 30 participants for further analysis. For the first set of analyses, we measured proportion of correct responses out of all trials. Adjusted $n_{p}^{2}$ values accompany each statistical test (Mordkoff, 2019).

### 3.1 ISI calibration procedure

**Figure 3:**
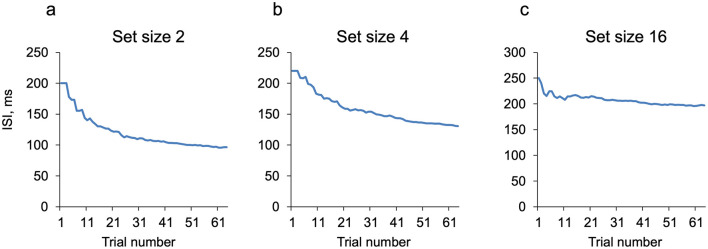
Calibration procedure data. Participants completed a 64-trial staircase procedure for each search goal set size at the start of the search task. **(a–c)** ISI changes throughout the course of the procedure, by set size.

**Figure 4:**
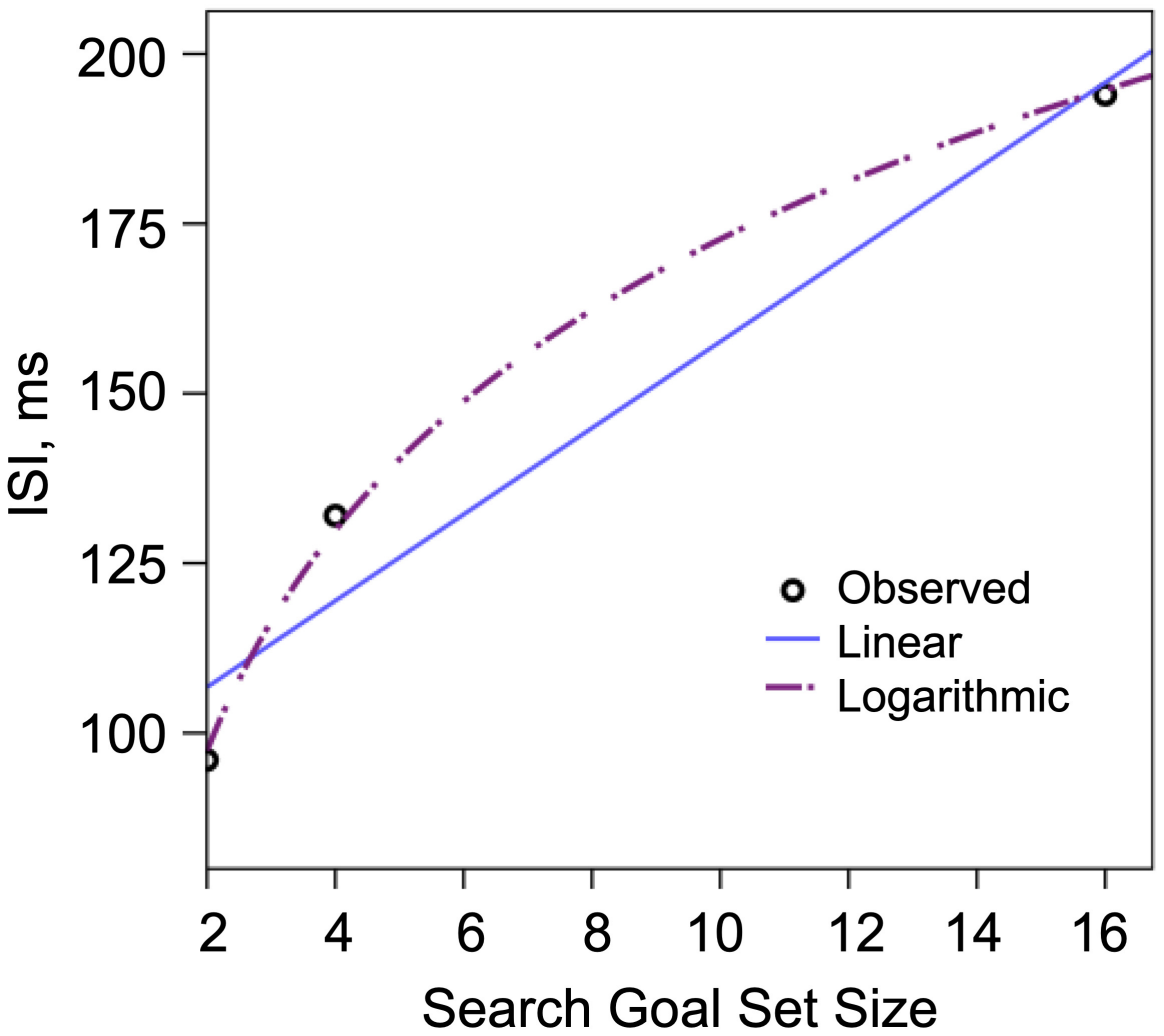
Final ISI plotted as a function of set size. Logarithmic and linear estimates are plotted; the logarithmic estimate better fits the data than does the linear estimate.

Overall, the calibration procedure was successful, yielding similar performance across set sizes in the no distraction (target alone) conditions during the main experiment (set size 2: *M* = 0.725, SD = 0.14; set size 4: *M* = 0.721, SD = 0.115; set size 16: *M* = 0.702, SD = 0.194; see Figure 3 for results from the calibration procedure). A one-way ANOVA found no significant difference across set sizes: *F*_(2, 58)_ = 0.225, *p* = 0.799, adj. $n_{p}^{2}$ = −0.027. Next, we fitted the ISI data to both a linear model and a logarithmic model to assess which model better explained the data. Though there were only three set sizes as observations, the result was nonetheless clear—the ISIs more closely followed a log_2_ increase in search speed with set size [*F*_(1, 1)_ = 687, *p* = 0.024], rather than a linear increase [*F*_(1, 1)_ = 16.8, *p* = 0.152], in line with prior hybrid visual-memory results (Drew and Wolfe, 2014). The mean ISI values were as follows: set size 2—*M* = 96, *SD* = 50.7; set size 4—*M* = 132, *SD* = 57.5; set size 16—*M* = 194, *SD* = 67.4. See Figure 4 for the comparison of final ISIs across search goal set size, and how the linear and logarithmic functions fit these data.

### 3.2 Accuracy

Using just the lag 2 data, at which distraction effects were the largest, we performed a repeated measures ANOVA with the factors trial type (TA, SC, DC) and set size (2, 4, 16). There was a main effect of trial type, *F*_(2, 58)_ = 105.67, *p* < 0.001, adj. $n_{p}^{2}\;=$ 0.777, with highest performance in target alone trials (*M* = 0.716, SE = 0.017), followed by SC trials (*M* = 0.527, SE = 0.022), and then DC trials (*M* = 0.416, SE = 0.018). There was also an interaction between set size and trial type *F*_(4, 116)_ = 2.53, *p* = 0.045, adj. $n_{p}^{2}\;=$ 0.049. These results suggested different attentional capture effects across set size. There were not significant differences across set size, which is by design due to the calibration procedure (set size 2 *M* = 0.569, SE = 0.024; set size 4 *M* = 0.552, SE = 0.018; set size 16 *M* = 0.528, SE = 0.030).

To investigate contingent capture, we performed an ANOVA assessing contingent attentional capture with the factors trial type (TA, SC) and set size (2, 4, 16). There was a main effect of trial type *F*_(1, 29)_ = 123.5, *p* < 0.001, adj. $n_{p}^{2}\;=$ 0.803, replicating contingent attentional capture, as well as an interaction between set size and trial type *F*_(2, 58)_ = 3.49, *p* = 0.041, adj. $n_{p}^{2}\;=$ 0.077, with the greatest attentional capture at set size 16 and the smallest at set size 2. *Post-hoc* tests revealed that contingent attentional capture was significant at each set size. Set size 2: *t*_(29)_ = 4.58, *p* < 0.001, *d* = 0.837, set size 4: *t*_(29)_ = 7.53, *p* < 0.001, *d* = 1.37, and set size 16: *t*_(29)_ = 7.71, *p* < 0.001, *d* = 1.41.

To investigate set-specific capture, we performed an ANOVA with the factors trial type (SC, DC) and set size (2, 4, 16). There was a main effect of trial type *F*_(1, 29)_ = 21.95, *p* < 0.001, adj. $n_{p}^{2}\;=$ 0.411, replicating set-specific capture, as well as an interaction between set size and trial type *F*_(2, 58)_ = 4.48, *p* = 0.015, adj. $n_{p}^{2}\;=$ 0.104, with the largest set-specific capture effect at set size 2 and the smallest at set size 16. While set-specific capture was significant at set size 2 [*t*_(29)_ = 5.73, *p* < 0.001, *d* = 1.05] and set size 4 [*t*_(29)_ = 2.79, *p* = 0.009, *d* = 0.509], it was not significant at set size 16 [*t*_(29)_ = 0.889, *p* = 0.38, *d* = 0.162].

**Figure 5:**
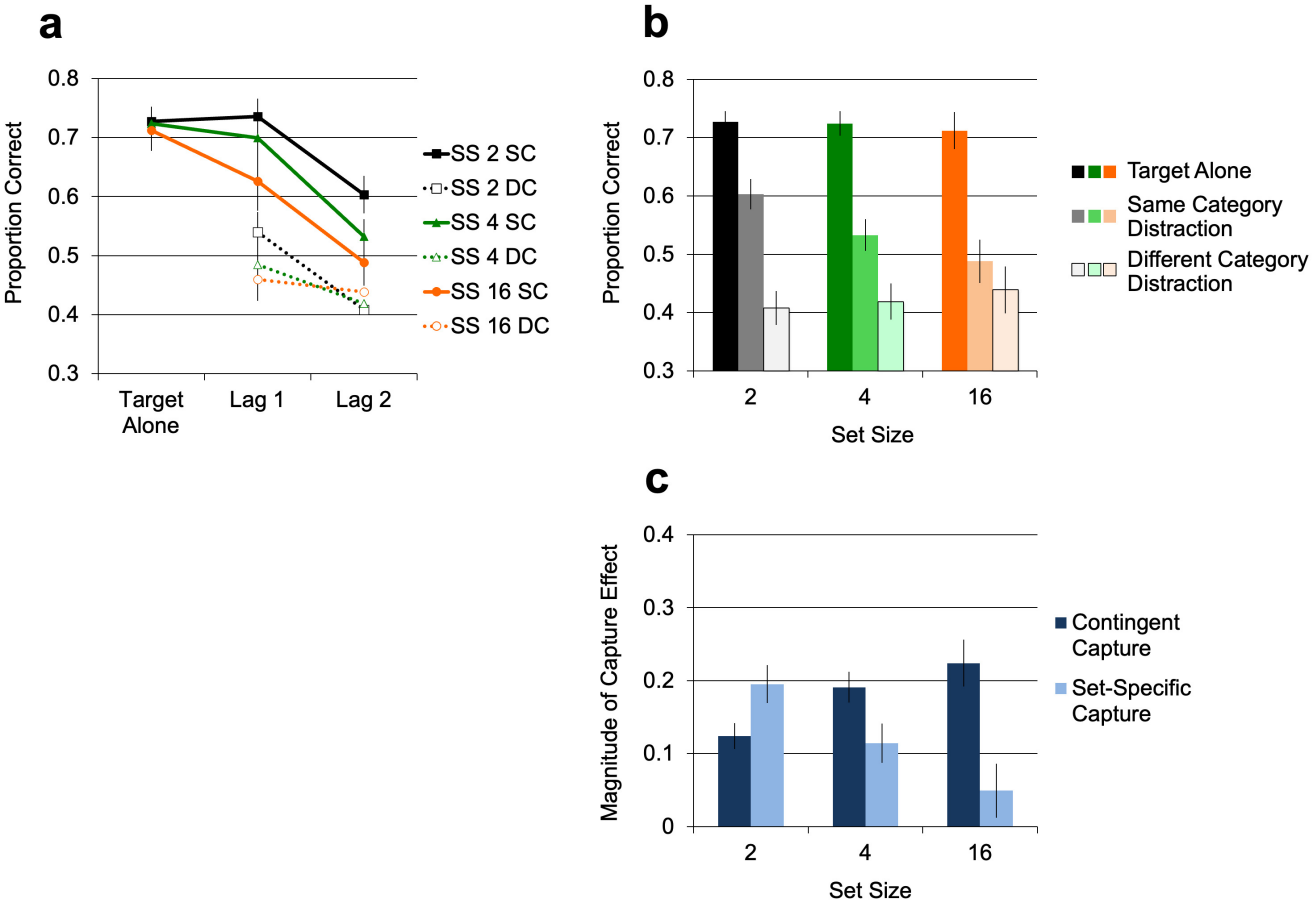
Proportion correct across all conditions. **(a)** Proportion correct responses to targets in target alone trials as well as lags 1 and 2 for both distractor conditions, across all set sizes. Different colors represent individual set sizes, and dotted lines represent the “different category” distraction trials (“same category” distraction plotted in solid lines). **(b)** Target alone, same category, and different category trial types by set size, using lag 2 data only. **(c)** Magnitude of contingent attentional capture and set-specific capture, plotted by set size, using lag 2 data only. Error bars represent standard error of the mean.

Finally, we examined lag effects for set-specific capture in a repeated measures ANOVA with the factors trial type (SC, DC), set size (2, 4, 16), and lag (1, 2). There was a main effect of lag *F*_(1, 29)_ = 41.39, *p* < 0.001, adj. $n_{p}^{2}\;=$ 0.574, showing that distraction effects were larger at lag 2 than at lag 1. There was also a main effect of trial type *F*_(1, 29)_ = 52.89, *p* < 0.001, adj. $n_{p}^{2}\;=$ 0.634, indicative of set-specific capture across both lags. There was also an interaction between trial type and lag, indicating more “lag-1 sparing” in SC trials than in DC trials *F*_(1, 29)_ = 10.20, *p* = 0.003, adj. $n_{p}^{2}\;=$ 0.235. Finally, there was a three-way interaction among set size, trial type, and lag: the difference in lag-1 sparing across SC and DC trials varied across set sizes, with the biggest difference in set size 2, and the smallest in set size 16, *F*_(2, 58)_ = 4.49, *p* = 0.016, adj. $n_{p}^{2}\;=$ 0.104 (see Figure 5).

Collectively, these results indicate that the magnitude of contingent attentional capture effects increases with search set size while the magnitude of set-specific capture effects decreases. Poorer performance in the SC condition with increased search set size drives these effects. The following analyses investigate whether these findings were due to increases in search set size or to various confounds.

### 3.3 Reaction time

As is the case in most RSVP tasks including those measuring the Attentional Blink (Hommel and Akyürek, 2005; Nielsen and Andersen, 2011; Raymond et al., 1992; Visser et al., 1999), attentional capture in dynamic displays (Folk et al., 2002; Moore et al., 2018; Moore and Weissman, 2010, 2011; Serences et al., 2005), and searching through memory using dynamic displays (Drew et al., 2014; Drew and Wolfe, 2014), accuracy was our primary dependent variable of interest. However, we recorded reaction time as an exploratory measure, keeping in mind that participants were told to emphasize accuracy and not to concern themselves with speed.

**Figure 6:**
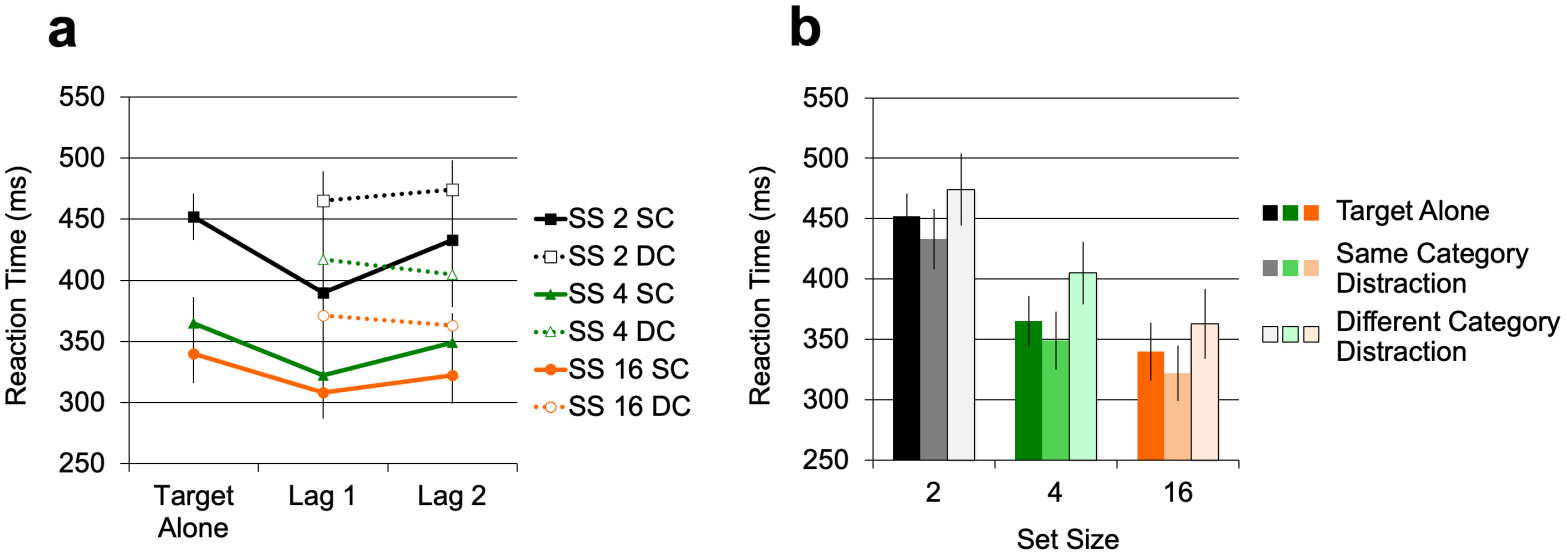
Reaction time to correctly identified targets, in milliseconds. **(a)** Reaction time for Target Alone trials as well as lags 1 and 2 for both distractor conditions, across set sizes. Different colors represent the individual set sizes, and dotted lines represent the different category trial type (same category trial type is plotted in a solid line). **(b)** Target alone, same category, and different category trial types by set size, using lag 2 data only.

Mirroring the accuracy analyses, with reaction time (in milliseconds) as the dependent variable, we conducted a 3 × 3 ANOVA using lag 2 data with the factors trial type (TA, SC, and DC) and set size (2, 4, 16) for trials that were correctly responded to. There was a main effect of trial type, with fastest responses to SC trials (M = 368, SE = 23), followed by TA trials (M = 386, SE = 17), and then DC trials (M = 414, SD = 23), *F*_(2, 58)_ = 11.63, *p* < 0.001, adj. $n_{\text{p}}^{2}$ = 0.286. *Post-hoc* tests demonstrate that the difference between each condition is significant. For SC < TA, *t*_(29)_ = 2.48, *p* = 0.019, *d* = 0.132. For TA < DC, *t*_(29)_ = 2.63, *p* = 0.014, *d* = 0.210. For SC < DA, *t*_(29)_ = 4.35, *p* < 0.001, *d* = 0.342. Faster responses to SC trials compared to TA and DC trials indicates evidence of attentional set enhancement. There was also a main effect of set size, with faster RTs to set size 16 (M = 342, SE = 23), followed by set size 4 (M = 373, SE = 23), followed by set size 2 (M = 453, SE = 22), *F*_(1, 29)_ = 15.02, *p* < 0.001, adj. $n_{\text{p}}^{2}$ = 0.341. *Post-hoc* tests confirm that the difference between set size 2 and set size 4 is significant *t*_(29)_ = 3.72, *p* = 0.002, *d* = 0.59, but the difference between set size 4 and set size 16 is not, *t*_(29)_ = 1.59, *p* = 0.121, *d* = 0.232 (see Figure 6).

To examine lag effects, we also performed a 2 × 2 ANOVA on RTs using trials with correct responses, with the factors trial type (SC, DC), set size (2, 4, 16), and lag (1, 2). As in the previous ANOVA, we found main effects of set size, *F*_(2, 58)_ = 13.26, *p* < 0.001, $n_{p}^{2}\;=$ 0.314, and trial type, *F*_(1, 29)_ = 47.63, *p* < 0.001, adj. $n_{p}^{2}\;=$ 0.622, with the same pattern observed above (faster RTs for larger set sizes and faster RTs for SC than DC trials). There was also a significant interaction between lag and trial type, *F*_(1, 29)_ = 7.77, *p* = 0.009, adj. $n_{p}^{2}$ = 0.211. SC trials were faster at lag 1 than lag 2, *t*_(29)_ = 3.46, *p* = 0.003, *d* = 0.202, whereas DC trials were non-significantly slower at lag 1 than lag 2, *t*_(29)_ = 0.417, *p* = 0.68, *d* = 0.027, consistent with the accuracy data that showed lag-1 sparing limited to SC trials.

### 3.4 Assessment of potential confounds

First, we examined whether participants were likely responding to distractor items that preceded targets rather than to targets themselves. A participant would respond to distractors instead of targets if their memory representation of the target were imprecise, allowing distractor items to be confused with targets. One possibility is that in spite of the memory test at the beginning of the block, the representations for targets in larger set sizes (16) were weaker, or less precise, than target representations in smaller set sizes (2). If this was the case, the set size-dependent effects reported above could be due to differences in how precisely the search sets were maintained, rather than the size of the search set.

The design of the task and the stimuli on SC and DC trials allowed us to examine this possibility. Critical distractors always faced the opposite direction from targets. For example, if the target was a laptop facing to the *right*, the critical distractor prior to that target was a distractor laptop facing to the *left* in the SC condition. This means that in the previous analyses, any response to a distractor would be marked as incorrect. However, because of the continuous RSVP display (i.e., no discrete trial breaks), participants were sometimes unaware that a target had appeared at all. Thus, incorrect scores could also be caused by a miss (i.e., did not respond). The prior analyses grouped both of these types of errors—misses and wrong responses—together. The following analysis isolates the incorrect responses from all other trials. By examining these trials, we can see how often participants mistakenly responded to the distractor rather than the target (thus causing an incorrect response), and whether this type of response varied with set size and trial type.

**Figure 7:**
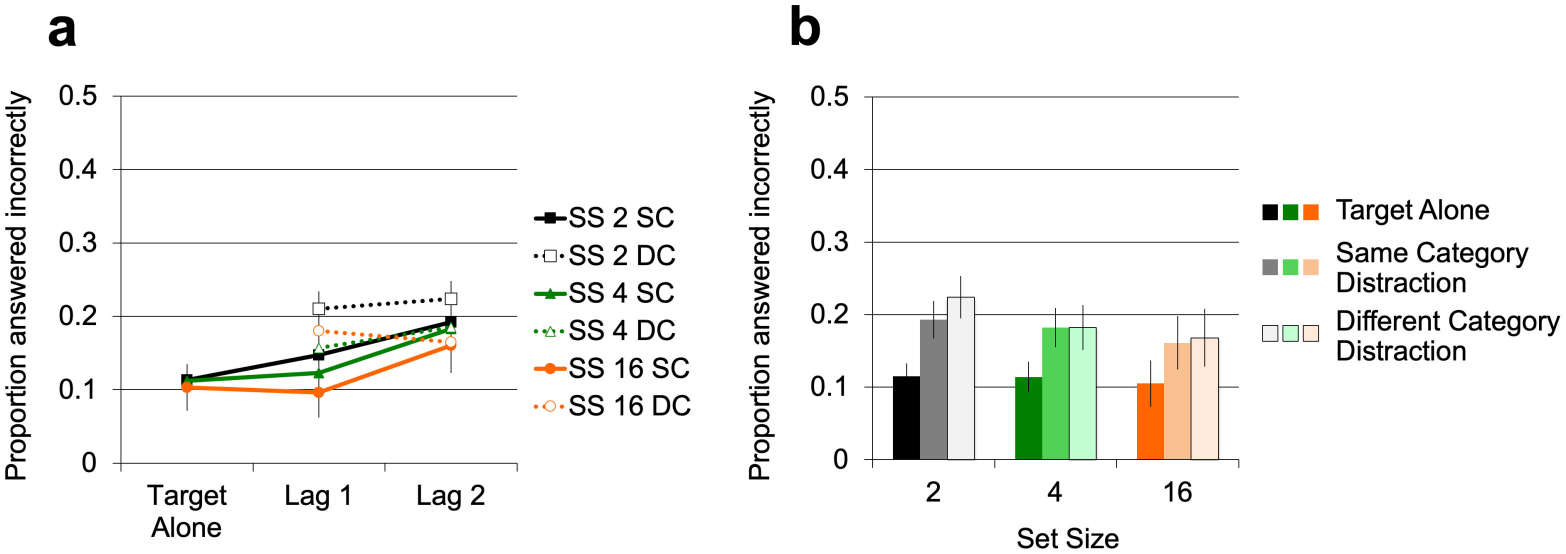
Proportion of targets responded to incorrectly. **(a)** Proportion responses to targets in target alone trials as well as lags 1 and 2 for both distractor conditions, across all set sizes. Different colors represent individual set sizes, and dotted lines represent the “different category” distraction trials (“same category” distraction plotted in solid lines). **(b)** Target alone, same category, and different category trial types by set size, using lag 2 data only. Error bars represent standard error of the mean.

We conducted an ANOVA with the factors trial type (TA, SC, DC) and set size (2, 4, 16) on just the lag 2 data, using proportion of incorrect responses (out of all trials) as the dependent variable. There was a main effect of trial type, *F*_(2, 58)_ = 15.12, *p* < 0.001, adj. $n_{p}^{2}\;=$ 0.320, because participants were more likely to respond incorrectly on trials with distraction (SC and DC) than on trials without distraction (TA), indicating that participants did sometimes respond to distractors. However, there was no main effect of set size, nor an interaction between set size and trial type (both Fs < 1). An ANOVA with the factors trial type (SC, DC), set size (2, 4, 16) and lag (1, 2) yielded null results as well (all Fs < 1). See Figure 7 for graphs of these response types.

This pattern of results indicates that responses to distractors did not contribute to the primary findings of differences in capture effects depending on search goal set size. In particular, if the memory traces for target items in set size 16 were weaker than the memory traces for targets at smaller set sizes, we would have expected more incorrect responses at this larger set size (and more for set size 4 than set size 2), because trials with incorrect responses are indicative that participants are mistaking distractors for targets. This was not the case. In fact, there were non-significantly more error responses in set size 2 on SC and DC trials (*M* = 0.208) than there were in set sizes 4 (*M* = 0.182) and 16 (*M* = 0.164). Moreover, a weaker memory trace should take longer to respond to, but we found that reaction times to targets were actually faster for larger set sizes than smaller ones.

Considering that miss trials may represent the purest form of attentional capture caused by distraction (as opposed to a decision-making failure due to a weak memory representation), we re-ran the primary analyses on lag 2 data removing incorrect responses from the total, so that only misses were considered errors. Each of the results mirrored our original analyses. In an ANOVA assessing contingent attentional capture with the factors trial type (TA, SC) and set size (2, 4, 16), there was a main effect of trial type, *F*_(1, 29)_ = 26.48, *p* < 0.001, adj. $n_{p}^{2}$ = 0.469, indicating contingent attentional capture, as well as an interaction between set size and trial type, *F*_(2, 58)_ = 6.04, *p* = 0.004, adj. $n_{p}^{2}$ = 0.168, once again with the greatest attentional capture at set size 16 and the smallest at set size 2. *Post-hoc* tests revealed that contingent attentional capture was significant at each set size (set size 2: [*t*_(29)_ = 2.15, *p* = 0.04, *d* = 0.386, set size 4: *t*_(29)_ = 4.30, *p* < 0.001, *d* = 0.772, and set size 16: *t*_(29)_ = 4.89, *p* < 0.001, *d* = 0.878]). In the ANOVA assessing set-specific capture with the factors trial type (SC, DC) and set size (2, 4, 16), there was a main effect of trial type, *F*_(1, 29)_ = 9.32, *p* = 0.005, adj. $n_{p}^{2}$ = 0.237. There was also an interaction between set size and trial type, *F*_(2, 58)_ = 3.95, *p* = 0.025, adj. $n_{p}^{2}$ = 0.116. *Post-hoc* tests revealed that set-specific capture was significant at set size 2 [*t*_(29)_ = 4.24, *p* < 0.001, *d* = 0.761] and set size 4 [*t*_(29)_ = 2.53, *p* = 0.017, *d* = 0.454], but not at set size 16 [*t*_(29)_ = 0.301, *p* = 0.77, *d* = 0.054]. Taken together, we reject the possibility that the results are due to stronger memory representations of targets at smaller set sizes than larger set sizes.

Another confound in the current design is that the timing of the display was different at each set size in order to compare this investigation to other hybrid searches and to avoid ceiling and floor performance. Thus, it is possible that the observed results reflect a difference in timing between contingent attentional capture (search goal enhancement) and set-specific capture, rather than an effect of set size on search goal enhancement. This would mean that set-specific capture may persist for an extended period (DC performance was consistent across set size), whereas the enhancement effect occurring from contingent attentional capture may dissipate quickly, a result consistent with (Moore and Weissman 2010).

To test this possibility, we examined contingent attentional capture effects according to ISI speed for each participant and set size, to see whether a slower speed was associated with less search goal enhancement (i.e., a greater attentional capture effect). For each set size, there was a non-significant positive correlation between the ISI selected for that participant-set size combination and the contingent attentional capture effect observed in that participant-set size combination (set size 2: *r*^2^ = 0.18, *p* = 0.35; set size 4: *r*^2^ = 0.13, *p* = 0.49; set size 16: *r*^2^ = 0.24, *p* = 0.20). Given that none of the correlations were significant, these results speak against timing differences in contingent attentional capture and set-specific capture as the only factor driving the difference in SC trial performance across set size. That said, the correlations were all positive, and there were only 30 participants in the study, a relatively small number for assessing null effects of correlations. Further studies using more lag timings or a consistent presentation speed across set size may elucidate differences in the time courses of set-specific capture and contingent attentional capture.

The timing confound could lead to another possible interpretation of the current results, which is that the process of rejecting a distractor is more difficult at larger set sizes, which may result in missing the target item. In this explanation, a more difficult rejection process may take longer, potentially causing more trials on which participants miss the subsequent target in the SC condition. Our reaction time data provide evidence against this explanation, because reaction times are actually longer for smaller set sizes than larger ones. Moreover, without a significant interaction between trial type and set size, there is no evidence that the distractor rejection mechanism is taking relatively longer at any particular set size.

Another possibility is that the enhancement effect varies not due to the number of search goals concurrently maintained, but with how much experience the participant has with the distractor stimulus and/or its related search goal, with more experience leading to a larger enhancement effect. Participants saw each individual target item (and the related distractors) more often in the set size 2 block than in the set size 16 block, even though the set size 2 block was shorter. Moreover, more experience with DC trials leads to a smaller set-specific capture effect (Moore and Wiemers, 2018), suggesting that experience can modulate capture effects. To assess this possibility, we compared whether the first half and second half of trials within a search set block differed in terms of SC performance, and SC performance in relation to other conditions. If the enhancement effect grows from the first half of the experiment to the next (and especially in set size 2, which has the most target repetitions), the experience may play a role in the observed set size effects. In a repeated measures ANOVA with the factors phase (first half, second half), set size (2, 4, 16) and trial type (TA, SC, DC), the critical three-way interaction was not significant, *F*_(4, 116)_ = 1.38, *p* = 0.255, adj. $n_{p}^{2}\;=$ 0.013. Neither was the interaction between trial type and phase, *F*_(2, 58)_ = 1.32, *p* = 0.275, adj. $n_{p}^{2}\;=$ 0.011, indicating that performance was not changing in a meaningful way across the first and second halves of the experiment. In an ANOVA that just focused on SC trials with the factors phase (first half, second half) and set size (2, 4, 16), the interaction between set size and phase was also not significant, *F*_(2, 58)_ = 0.188, *p* = 0.829, adj. $n_{p}^{2}\;=$ 0.028. Thus, it is unlikely that the observed enhancement effect difference across search set size is related to overall experience with the search items. Similarly, (Guild et al. 2014) found that how much exposure participants had to targets did not affect search.

## 4 Discussion

In the present study, we investigated how attentional capture effects, such as contingent attentional capture and set-specific capture effects, change as multiple search goals are concurrently maintained in memory. Principally, we were interested in how search goal enhancement changed as the concurrent search goals scaled up. There were four main findings. First, we found that search goal enhancement is flexible to task demands. Contingent attentional capture effects were greater when there were many search goals than when there were few search goals, but set-specific capture effects showed the opposite pattern. This effect was driven by the SC condition. This supports a model of search goal enhancement that is flexible to task demands, because the number of concurrent search goals causes the goals to be held in different memory stores (Duan et al., 2025; Saltzmann et al., 2024), and challenges an “all-or-nothing” view of enhancement (Moore and Weissman, 2014). Second, we found lag-1 sparing, an effect in which target processing is relatively better when it immediately follows a distractor item in an RSVP display, as opposed to when it is separated from the distractor by one frame (Dux et al., 2014). Third, we replicated prior findings from hybrid visual-memory search tasks, including a log-linear increase in search speed as the number of search goals increases (Drew and Wolfe, 2014; Wolfe, 2012). Finally, we successfully replicated prior contingent attentional capture and set-specific capture findings using object stimuli, with critical distractors originating from the same category as the search targets.

As contingent attentional capture effects increased with the number of concurrently maintained search goals in the present study, set-specific capture effects decreased. These effects were driven by lower SC performance with increased set size, while DC and Target Alone performance remained largely consistent across set size. For example, your search for *last week's* broccoli became more difficult as you searched for more ingredients simultaneously and were distracted by today's broccoli. As you maintain more concurrent search goals, the location of maintenance changes due to the task demands. Where in memory search goals are held is an active area of research (Berggren and Eimer, 2016; Duan et al., 2025; Fratescu et al., 2019; Giammarco et al., 2016; Olivers et al., 2011; Saltzmann et al., 2024; Woodman et al., 2013; Wyble et al., 2013). With a small set size, the search goals can be maintained in VWM (Duan et al., 2025; Zhou et al., 2020), with quick access to the focus of attention, and thus are subjected to robust goal enhancement. However, larger set sizes require ALTM for maintenance (Saltzmann et al., 2024), with more difficult access to the focus of attention, resulting in less effective goal enhancement. See (Plater et al. 2020) for an alternative view, in which it may not be necessary for ALTM to house these representations.

In our proposed mechanisms of contingent and set-specific capture, the first step is the same—attention is captured by a distractor that bears resemblance to a current search goal, and this item is selected for further, serial processing. At the same time, the related search goal is enhanced to the focus of attention (Moore and Weissman, 2010, 2011). This process would be quick if the number of concurrent search goals was small, as they could be held in VWM with easy access to the focus of attention (Duan et al., 2025; Zhou et al., 2020). However, larger memory set sizes would need to be maintained elsewhere, perhaps in ALTM, which would take longer to access the focus of attention (Duan et al., 2025; Saltzmann et al., 2024). As concurrently maintained search goals scale up and change the memory store that they are held in, the search goal related to the distractor will take longer to be enhanced to the focus of attention. This resulted in decreasing accuracy on SC trials at higher set sizes compared to lower set sizes, which drove an increase in the magnitude of the contingent attentional capture effect and a decrease in the magnitude of the set-specific capture effect at higher memory set sizes. This flexibility of goal enhancement depended both on the number of concurrently maintained search goals and the location of their maintenance challenges the previous uniform “all-or-nothing” view of enhancement (Moore and Weissman, 2014).

Previously, Moore and colleagues demonstrated that although multiple search goals could be maintained at one time, only a single goal could be enhanced at a time (Moore and Weissman, 2010, 2011, 2014). The mechanism underlying this enhancement appeared to be automatic: attending to a distractor or target would cause the related search template to enter a limited-capacity focus of attention for further processing, effectively enhancing that search template compared to the other concurrently maintained sets. If this enhancement is an automatic process, it should happen uniformly each time a new search template is enhanced. Because the focus of attention can only enhance a single goal at a time, this limited capacity produces a bottleneck in processing (Moore and Weissman, 2014). However, further work showed that experience with a memory set could reduce the set-specific capture effects for that set (Moore and Wiemers, 2018), suggesting that there are circumstances under which the enhancement process changes due to task demands. Similarly, the current study suggests that goal enhancement changes due to task demands, in this case due to the number of concurrent search goals.

Several follow-up analyses addressed confounds and produced findings that ruled out alternative explanations for the change in SC performance across set size. First, there was no effect of set size on incorrect responses (i.e., responding to the distractor that appeared before the target). Moreover, the overall pattern of results remained unchanged when only considering misses as errors (i.e., removing incorrect responses). This indicates that the general pattern of results, and in particular the change in SC performance with set size, was not due to participants' responding to the distractor item (which always faced opposite the target) instead of the target. In other words, it does not appear that participants confused distractors with targets *more often* in the larger set sizes than in the smaller ones. Moreover, reaction times were not longer at larger set sizes than smaller ones (in fact the opposite was observed), suggesting that participants were not engaging in a lengthier decision process due to weaker memory traces at larger set sizes. These results suggest that memory precision did not differ due to set size, and that memory precision cannot account for the current results. This is in line with evidence that long-term memory can be incredibly precise (Brady et al., 2008; Chung et al., 2023), especially when participants are repeatedly exposed to the targets (Miner et al., 2020). In the current study, participants first learned their targets, followed by an initial memory test, before the visual search began. Participants consistently demonstrated high accuracy for their targets on the memory test, regardless of set size.

Another analysis addressed the issue of the timing confound with set size, investigating the possibility that the enhancement effect operates on a different timescale than set-specific capture. We found no significant correlation between the interstimulus interval selected for the particular participant and set size, and SC performance or set-specific capture. That said, it is notable that every correlation in this analysis was positive, which is the direction that would be predicted if goal enhancement dissipates faster than do the costs of set-specific capture. Future investigations could elucidate this issue by including more “lag” timings in a similar experiment (e.g., such as lag 3, lag 4, etc.) so that the time course of search goal enhancement by set size can be mapped more precisely.

A similar study found results consistent with these while also addressing timing confounds. In a series of five experiments, Drew and colleagues implemented an attentional blink paradigm to examine the mechanisms of hybrid visual search (Drew et al., 2014). They required participants to search for 2, 4, 8, or 16 objects in an RSVP of 16 items. The first target in the RSVP display (T1) was always one of these memorized objects. The second target (T2) was either a colored letter or an object (not in the search set), framed in red. The authors examined performance for identifying T2 when T1 was identified. In drawing a parallel to the present study, T1 is like the distractor in SC and DC conditions, and T2 is like the target. In Drew and colleagues' experiments, the task of identifying T2 was always distinct from T1, and T2 was a different target type. Thus, participants knew in advance of the trial that they had to switch from one set of search goals to a different type of search on each trial (e.g., “search for the memorized objects for T1; now search for the red framed object for T2”). In some sense, this task is similar to DC trials in the present study, because a switch of search goals from T1 to T2 is required. On the other hand, in the present study the participant cannot predict when a DC trial will occur. Another difference is that in the Drew et al. study, there was no test of an enhancement effect. In no condition across the five experiments was the T2 task similar enough to the T1 search goal in order to draw a parallel to SC trials. Nonetheless, the authors found that the attentional blink magnitude did *not* vary with set size unless T1 processing had to be completed “online,” or the T1 was well masked (conditions not met in the present investigation). As a parallel, DC performance did not differ in the present study across set size, for similar reasons as in (Drew et al. 2014).

The ISI adjustment in the current study also introduces a confound with the attentional blink. At lower set sizes, the ISI is quicker, and situated in the appropriate timeframe for an attentional blink. However, at larger set sizes, the ISI is much slower, and situated outside of the typical attentional blink timeframe. Despite this difference, the mechanism of the attentional blink would likely be the same at both set sizes. It is likely that the attentional blink would operate more slowly at a larger set size due to the required memory search. Thus, the attentional blink would also be subject to task demands. This is in line with the (Drew et al. 2014) results, as they found similar attentional blinks at lag 2 across set sizes (2, 4, 8, and 16), even when the stimulus onset asynchrony increased to 201 ms in an RSVP stream of objects (Experiment 4). This is similar to the average ISI of 194 ms for set size 16 in the current study.

A final confound in the present results and study design was that participants had more experience with individual search goals in set size 2 than in set size 4 or especially set size 16. To examine whether this confound could explain the primary enhancement effect result, we measured performance across epochs of the experiment. There was no evidence to support the idea that enhancement effects vary with experience with a search goal, as performance was similar at different times within a block. With all of these alternative possibilities eliminated, the cause of the enhancement effect appears to be because search goal enhancement is only triggered for search goals that are held in VWM—which is capacity limited—and not for goals that are held in ALTM.

Though there were two “lag” timings in this experiment, the greatest distraction effects were visible at lag 2 while we observed a phenomenon known as lag-1 sparing. This is an effect in which target processing is relatively better when it immediately follows a distractor item in an RSVP display, as opposed to when it is separated from the distractor by one frame (Dux et al., 2014). The improved performance at lag 1 may be because the distractor and target are processed together. Lag-1 sparing was first identified as part of the attentional blink phenomenon (Dux et al., 2014; Hommel and Akyürek, 2005; Raymond et al., 1992; Zivony and Eimer, 2021). In attentional blink experiments, participants are required to identify two targets in a rapid serial visual presentation display. Detecting or identifying the second target is impaired when the first target is identified accurately and it appears soon before the second target. The shorter the lag (number of frames) between the first and second targets, the greater the attentional blink cost, with one exception. Most attentional blink studies find lag-1 sparing, in which performance is slightly better when the first target appears immediately ahead of the second, as opposed to being separated in time by at least one more frame/item. In the current study, performance was better at lag 1 than at lag 2, and this effect was larger in the SC condition than the DC condition, as well as larger for set size 2 than 16. This result, observed in both accuracy and reaction time, is in line with a review of lag-1 sparing detailing that lag-1 sparing occurs except when there is a shift of task or location (Visser et al., 1999). As we have argued above, the DC condition can be thought of as a “set switch” in that internal attention must shift from the search goal corresponding to the distractor to the search goal corresponding to the target. Previous set-specific capture experiments did not find lag-1 sparing, but these tasks required spatial shifts of attention (Moore and Weissman, 2010; Moore and Wiemers, 2018).

Our present experiment used a similar method as (Drew and Wolfe 2014) to assess hybrid search, and our findings replicated theirs of a log-linear increase in search speed as the number of search goals increases (Cunningham and Wolfe, 2014; Drew et al., 2016; Wolfe, 2012). In the present study, the calibration procedure determined the appropriate presentation speed in order to ensure equal accuracy across set size in the “target alone” (no distraction) condition in the main experiment. While we tested only three set sizes, the results still suggested a logarithmic (log_2_) rather than a linear change of search speed with the increase in search goals, replicating other hybrid search findings. Previous hybrid search studies have examined four or five set sizes and sometimes included a condition in which 100 targets were searched for at a time. Future research could include more set sizes to provide a better estimate of the search speed function with set-specific capture.

One difference, however, from (Drew and Wolfe 2014) is that overall performance was lower in the present study. This discrepancy is likely due to the nature of the present task. We used a continuous display, rather than discrete trials interrogating a response on every trial. With discrete trials, chance performance was at 50% (target “present” or “absent”). In the current study, participants first had no warning or indication about when a target *might* occur. Moreover, participants were required to identify the direction the target item was facing once it was identified, a higher demand than merely detecting its presence. Though this change in task yielded a difference in overall performance, the search time increase with set size nonetheless followed the same pattern as prior studies.

The present results also replicate previous findings of contingent attentional capture and set-specific capture, using objects as target and distractor stimuli for the first time to assess set-specific capture. Some prior studies that have employed objects in attentional capture settings have used the memorized objects as distractors (Giammarco et al., 2016). In others, contingent attentional capture has been measured using target-resembling distractors from the same object category (Lim et al., 2021; Seidl-Rathkopf et al., 2015; Wyble et al., 2013). In the present study, both contingent attentional capture and set-specific capture effects were found with objects from the same category used as distractors; these varied from the target on a number of different factors. This result fits with prior data from visual search studies that have used real objects as stimuli, and have measured the existence of perceptual and conceptual relatedness (Biggs et al., 2015; Lim et al., 2021; Mitroff et al., 2015; Seidl-Rathkopf et al., 2015). Future research should continue to leverage the ability to measure both capture effects using object stimuli.

## Data Availability

The datasets presented in this study can be found in online repositories. The names of the repository/repositories and accession number(s) can be found in the article/supplementary material.

## Footnotes

1 For each category, we tested several research assistants and used only object categories that we could agree upon as facing in the same direction. However, it is possible that object affordances may interfere with these judgments (Cho and Proctor, 2013).

2 The speed change at each reversal in order, in ms, was: 40, 20, 20, 10, 10, 5. Any subsequent reversals that occurred were also 5 ms.
