# Correction: Attentional capture effects are modulated by the number of concurrently maintained search goals

**DOI:** 10.3389/fcogn.2026.1878705

**Published:** 2026-06-02

**Authors:** Katherine Sledge Moore, Ariel M. Kershner

**Affiliations:** 1 Department of Psychology, Arcadia University, Glenside, PA, United States; 2 Department of Psychology, Neumann University, Aston Township, PA, United States

**Keywords:** attentional capture, hybrid search, memory, RSVP, set-specific capture, visual search

There were two incorrect statistics in the original publication. In the original, *p* = 0.27 was listed twice for two statistics, whose true values were actually *p* = 0.019 and *p* = 0.014. The original sentence read: For SC < TA, *t*_(29)_ = 2.48, *p* = 0.27, *d* = 0.132. For TA < DC, *t*_(29)_ = 2.63, *p* = 0.27, *d* = 0.210. For SC < DA, *t*_(29)_ = 4.35, *p* < 0.001, *d* = 0.342.

A correction has been made to the Section 3.3 *Reaction time*, paragraph 2:

“Mirroring the accuracy analyses, with reaction time (in milliseconds) as the dependent variable, we conducted a 3 × 3 ANOVA using lag 2 data with the factors trial type (TA, SC, and DC) and set size (2, 4, 16) for trials that were correctly responded to. There was a main effect of trial type, with fastest responses to SC trials (M = 368, SE = 23), followed by TA trials (M = 386, SE = 17), and then DC trials (M = 414, SD = 23), *F*_(2, 58)_ = 11.63, *p* < 0.001, adj. $n_{\text{p}}^{2}$ = 0.286. *Post-hoc* tests demonstrate that the difference between each condition is significant. For SC < TA, *t*_(29)_ = 2.48, *p* = 0.019, *d* = 0.132. For TA < DC, *t*_(29)_ = 2.63, *p* = 0.014, *d* = 0.210. For SC < DA, *t*_(29)_ = 4.35, *p* < 0.001, *d* = 0.342. Faster responses to SC trials compared to TA and DC trials indicates evidence of attentional set enhancement. There was also a main effect of set size, with faster RTs to set size 16 (M = 342, SE = 23), followed by set size 4 (M = 373, SE = 23), followed by set size 2 (M = 453, SE = 22), *F*_(1, 29)_ = 15.02, *p* < 0.001, adj. $n_{\text{p}}^{2}$ = 0.341. *Post-hoc* tests confirm that the difference between set size 2 and set size 4 is significant *t*_(29)_ = 3.72, *p* = 0.002, *d* = 0.59, but the difference between set size 4 and set size 16 is not, *t*_(29)_ = 1.59, *p* = 0.121, *d* = 0.232 (see **Figure 6**).”

The original version of this article has been updated.

